# Oral paracoccidioidomycosis in a young male from northeastern Brazil, a non-endemic region^؆^

**DOI:** 10.1016/j.abd.2026.501311

**Published:** 2026-03-23

**Authors:** Gerhilde Callou Sampaio, Marianne de Vasconcelos Carvalho, Jéssica da Silva Cunha, Bruno Augusto Benevenuto de Andrade, Ricardo Alves Mesquita, José Alcides Almeida de Arruda

**Affiliations:** a Department of Oral and Maxillofacial Pathology, Faculdade de Odontologia, Universidade de Pernambuco, Recife, Pernambuco, Brazil; b Post-Graduation Program, Faculdade de Odontologia, Universidade de Pernambuco, Recife, Pernambuco, Brazil; c Integrated Center of Pathological Anatomy, Hospital Universitário Oswaldo Cruz, Universidade de Pernambuco, Recife, Pernambuco, Brazil; d Department of Oral Diagnosis and Pathology, Faculdade de Odontologia, Universidade Federal do Rio de Janeiro, Rio de Janeiro, Brazil; e Department of Oral Surgery, Pathology and Clinical Dentistry, Faculdade de Odontologia, Universidade Federal de Minas Gerais, Belo Horizonte, Minas Gerais, Brazil

*Dear Editor,*

Paracoccidioidomycosis (PCM), formerly known as South American blastomycosis, was first described in 1908 by Adolpho Lutz. It is a systemic mycosis caused by thermally dimorphic fungi of the *Paracoccidioides brasiliensis* complex or by *P. lutzii*.^1^ Importantly, PCM has not been included in the World Health Organization’s portfolio of neglected tropical diseases. Estimates suggest that nearly 10 million individuals in Latin America are infected, with PCM representing the leading cause of mortality among systemic mycoses.^2^ Its burden is likely underestimated, as compulsory reporting was only recently implemented in Brazil. Infection occurs through inhalation of conidia from soil, predominantly affecting rural populations.^1^, ^3^ The clinical spectrum ranges from acute to chronic forms, often involving multiple organs, including the oral cavity.^1^, ^3^, ^4^ This report describes a rare case of oral PCM in a young patient from a non-endemic region of northeastern Brazil.

A 37-year-old male farmer from Rio Formoso, Pernambuco, Brazil (latitude − 8.66146, longitude − 35.1516), presented with a painless oral lesion that had progressed over 12-months. The patient was a chronic smoker and alcohol user, with no significant personal or family medical history. Extraoral examination revealed no submandibular or cervical lymphadenopathy. He denied weight loss, cough, dyspnea, and fever. Intraoral examination revealed an ulcerated, moriform lesion with infiltrative borders on the right buccal mucosa, extending toward the labial commissure and measuring approximately 3 × 3 cm (Fig. 1). The differential diagnosis included oral leukoplakia and oral squamous cell carcinoma. Computed tomography of the thorax demonstrated subtle interstitial changes consistent with chronic disease in both lungs. Cranial and sinus imaging revealed no abnormalities. Laboratory tests showed normal biochemical parameters, with negative VDRL and HIV results.

**Fig. 1:**
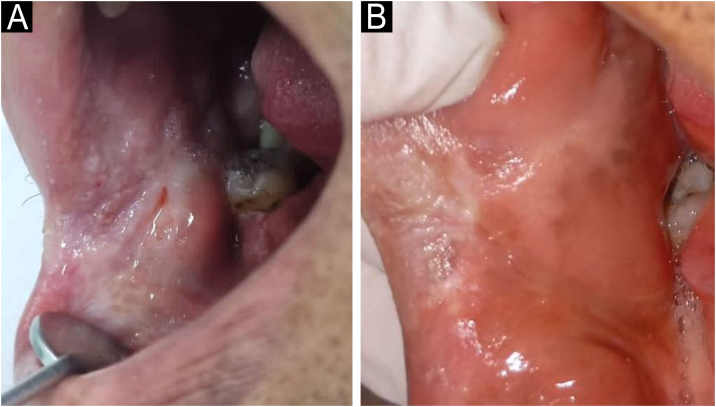
Clinical features of oral paracoccidioidomycosis. (A) Intraoral view showing an ulcerated, moriform lesion with infiltrative borders on the right buccal mucosa contiguous to the labial commissure, measuring approximately 3 × 3 cm. (B) Clinical follow-up image after 24-months demonstrating cicatricial fibrosis and no evidence of recurrence.

An incisional biopsy was performed. Histopathological examination revealed chronic inflammation with multinucleated giant cells, lymphocytes, and plasma cells within the connective tissue stroma. Round fungal structures consistent with *Paracoccidioides* spp. were identified. Grocott-Gomori Methenamine Silver (GMS) and Periodic Acid-Schiff (PAS) stains confirmed multiple budding yeasts in the characteristic “pilot wheel” arrangement (Fig. 2). A diagnosis of PCM was established. The patient was treated with oral itraconazole 200 mg/day for 12-months. No adverse effects were reported. At the 24-month follow-up, there was no clinical evidence of recurrence.

**Fig. 2:**
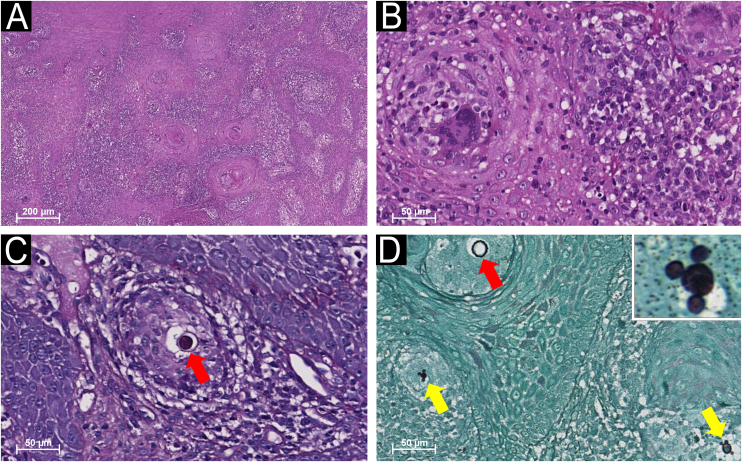
Histopathological features of oral paracoccidioidomycosis. (A) Low-power view showing pseudoepitheliomatous hyperplasia with underlying chronic inflammatory infiltrate. (B) Higher magnification demonstrating multinucleated giant cells, lymphocytes, and plasma cells. (C) Periodic acid-Schiff (PAS) and (D) Grocott-Gomori Methenamine Silver (GMS) staining illustrating multiple budding yeasts of the *Paracoccidioides* spp. in the profile of the “pilot wheel” arrangement (yellow arrows; inset) or round structures (red arrows) (hematoxylin and eosin staining: 40× and 200× magnification; PAS and GMS: 200× magnification).

Data from a literature review indicate that only eight cases of oral PCM in northeastern Brazil have been documented hitherto (Table 1).^5^, ^6^, ^7^, ^8^ Previous literature of autochthonous cases in Ceará and the high number of cases reported in specific regions of Maranhão suggest that humid areas of northeastern Brazil may also be considered endemic.^1^ Nonetheless, both the present case and the other patients with oral PCM from northeastern Brazil had no history of travel to recognized endemic zones.^5^, ^6^, ^7^, ^8^

**Table 1:** Data on cases of oral paracoccidioidomycosis diagnosed in individuals from Northeastern Brazil available in the PubMed, Scopus, Embase, LILACS, and Web of Science databases.

Study	Age/Sex and occupation	Clinical features	Differential diagnosis	Systemic manifestations	Evolution time	Diagnostic rendering	Treatment	Outcome (follow-up)
Façanha et al.,^5^ 2010; Ceará	44/M; agriculturist	Ulcero-nodular lesion on the oral mucosa adjacent to the right first molar	NA	Cervical lymphadenopathy, dry cough, fever, and weight loss	7-months	H&E and PAS	Itraconazole (200 mg)	Alive (3-months)
Mota et al.,^6^ 2019; Ceará	65/M; farmer	Hard and soft palate	Leishmaniasis	Cutaneous and nasal mucosal lesions; pulmonary infiltration; maxillo-ethmoidal sinusitis; deviated nasal septum to the left	36-months	H&E, PAS, GMS, culture, serology, and PCR	Itraconazole (200 mg)	Alive (33-months)
Souza et al.,^7^ 2019; Pernambuco	40/M	Buccal mucosa	OSCC and PCM	Pain	1.5-months	H&E, PAS, and GMS	NA	NA
	43/M	Tongue, soft palate, buccal mucosa, and floor of the mouth	OSCC	Pain and dysphagia	3-months	H&E, PAS, and GMS	NA	NA
	48/M	Hard palate	OSCC	Pain	0.7-months	H&E, PAS, and GMS	NA	NA
	65/M	Buccal mucosa and lower lip	Leishmaniasis, histoplasmosis, PCM, OSCC, and actinic cheilitis	Pain	4-months	H&E, PAS, and GMS	NA	NA
	75/M	Upper lip, maxillary alveolar ridge, and hard palate	OSCC and PCM	Pain	2-months	H&E, PAS, and GMS	NA	NA
Sousa et al.,^8^ 2021; Maranhão	68/M; farmer	Painful moriform ulcers in the hard and soft palate	OSCC and PCM	Toe lesions, weight loss, respiratory difficulty, physical debility	3-months	H&E, PAS, and GMS	Amphotericin B	Deceased (15 days)

**Note:** GMS, Grocott-Gomori Methenamine Silver; H&E, Hematoxylin and Eosin; M, Male; NA, Not Available; OSCC, Oral Squamous Cell Carcinoma; PAS, Periodic Acid-Schiff; PCM, Paracoccidioidomycosis.

We previously reported that oral PCM lesions accounted for 0.3% of specimens submitted for histopathological evaluation.^4^ Oral mucosal lesions may represent the first clinical manifestation of PCM, with approximately 60% of patients presenting with oral and/or oropharyngeal involvement,^9^ most frequently affecting the gingiva/alveolar ridge.^4^ Middle-aged and older men are disproportionately affected,^4^, ^7^ reflecting both agricultural exposure and the possible protective effect of β-estradiol in women; the latter inhibits the transition of the fungus to the pathogenic yeast form.^10^ Although the armadillo is acknowledged as a natural reservoir, neither zoonotic nor human-to-human transmission has been demonstrated.^3^

The diagnosis of PCM based on oral lesions is challenging, as they often emulate other infectious diseases and malignancies.^4^ Oral manifestations are typically ulcerative or erosive, multiple, erythematous, with granular surfaces and hemorrhagic petechiae, termed “moriform stomatitis”. The mechanism by which the fungus compromises oral tissues remains elusive.^4^ Diagnostic approaches include direct microscopy, culture, histopathology, and molecular methods.^1^, ^3^ Histopathology shows chronic inflammation with macrophages and multinucleated giant cells containing yeast cells. The fungi, larger than those observed in histoplasmosis, are demonstrable with GMS or PAS stains. A hallmark feature is the presence of multiple budding daughter cells radiating from the parent yeast, forming a “pilot wheel” appearance.^4^ Current Brazilian guidelines recommend itraconazole (200 mg daily for 9–12 months) as first-line therapy for mild-to-moderate disease.^3^

Awareness of the clinicopathological spectrum of PCM is therefore crucial for timely diagnosis, as its evolving ecoepidemiology and the probable expansion of autochthonous foci pose significant public health concerns.

## ORCID IDs

Gerhilde Callou Sampaio: 0000-0001-8437-471X

Marianne de Vasconcelos Carvalho: 0000-0002-6815-5696

Jéssica da Silva Cunha: 0000-0003-1570-2964

Bruno Augusto Benevenuto de Andrade: 0000-0002-3259-606X

Ricardo Alves Mesquita: 0000-0003-3207-4007

José Alcides Almeida de Arruda: 0000-0002-6599-3950

## Authors' contributions

Gerhilde Callou Sampaio: Conception and planning of the study; intellectual participation in the diagnosis and management of the case; drafting and editing of the manuscript; critical review of the manuscript.

Marianne de Vasconcelos Carvalho: Intellectual participation in the diagnosis of the case; critical review of the manuscript.

Jéssica da Silva Cunha: Intellectual participation in the diagnosis of the case; critical review of the manuscript.

Bruno Augusto Benevenuto de Andrade: Critical review of the literature; drafting and editing of the manuscript; critical review of the manuscript.

Ricardo Alves Mesquita: Intellectual participation in the diagnosis of the case; critical review of the manuscript.

José Alcides Almeida de Arruda: Critical review of the literature; intellectual participation in the diagnosis of the case; drafting and editing of the manuscript; critical review of the manuscript; final approval of the manuscript.

## Financial support

None declared.

## Research data availability

Does not apply.

## Conflicts of interest

None declared.
