# Supplementary material for: Oral paracoccidioidomycosis in a young male from northeastern Brazil, a non-endemic region
Source: An Bras Dermatol. 2026 Mar 23;101(2):501311. doi: 10.1016/j.abd.2026.501311 (PMC13049382; doi:10.1016/j.abd.2026.501311)
Supplement: Supplementary file 1 [file mmc1.docx]

**ABD-D-25-00646**

**Supplementary Material**

Searches were conducted in September 2025 using the following strategy: “(paracoccidioidomycosis OR “South American blastomycosis” OR “paracoccidioidal granuloma” OR “Lobo disease” OR “Lutz-Splendore-Almeida disease”) AND (“alveolar process” OR “alveolar ridge” OR “buccal mucosa” OR “buccal mucosal” OR “floor of the mouth” OR gingiva OR gingivae OR “hard palate” OR jaw OR jaws OR lip OR lips OR mandible OR mandibles OR maxilla OR maxillae OR mouth OR oral OR “oral cavity” OR “oral mucosa” OR “oral mucosae” OR oropharynges OR oropharynx OR palate OR perioral OR “soft palate” OR tongue OR tonsil OR tonsils) AND (Brazil OR northeast OR Maranhão OR Piauí OR Ceará OR “Rio Grande do Norte” OR Paraíba OR Pernambuco OR Alagoas OR Sergipe OR Bahia).

A total of 450 records were retrieved from electronic databases (PubMed: 138; Scopus: 82; Embase: 57; LILACS: 21, and Web of Science: 152), of which 4 articles comprising 8 cases were included in the qualitative analysis.
